# Supplementary material for: A single-particle analysis method for detecting membrane remodelling and curvature sensing
Source: J Cell Sci. 2024 Nov 7;137(21):jcs263533. doi: 10.1242/jcs.263533 (PMC11574359; doi:10.1242/jcs.263533)
Supplement: Supplementary information [file joces-137-263533-s1.pdf]

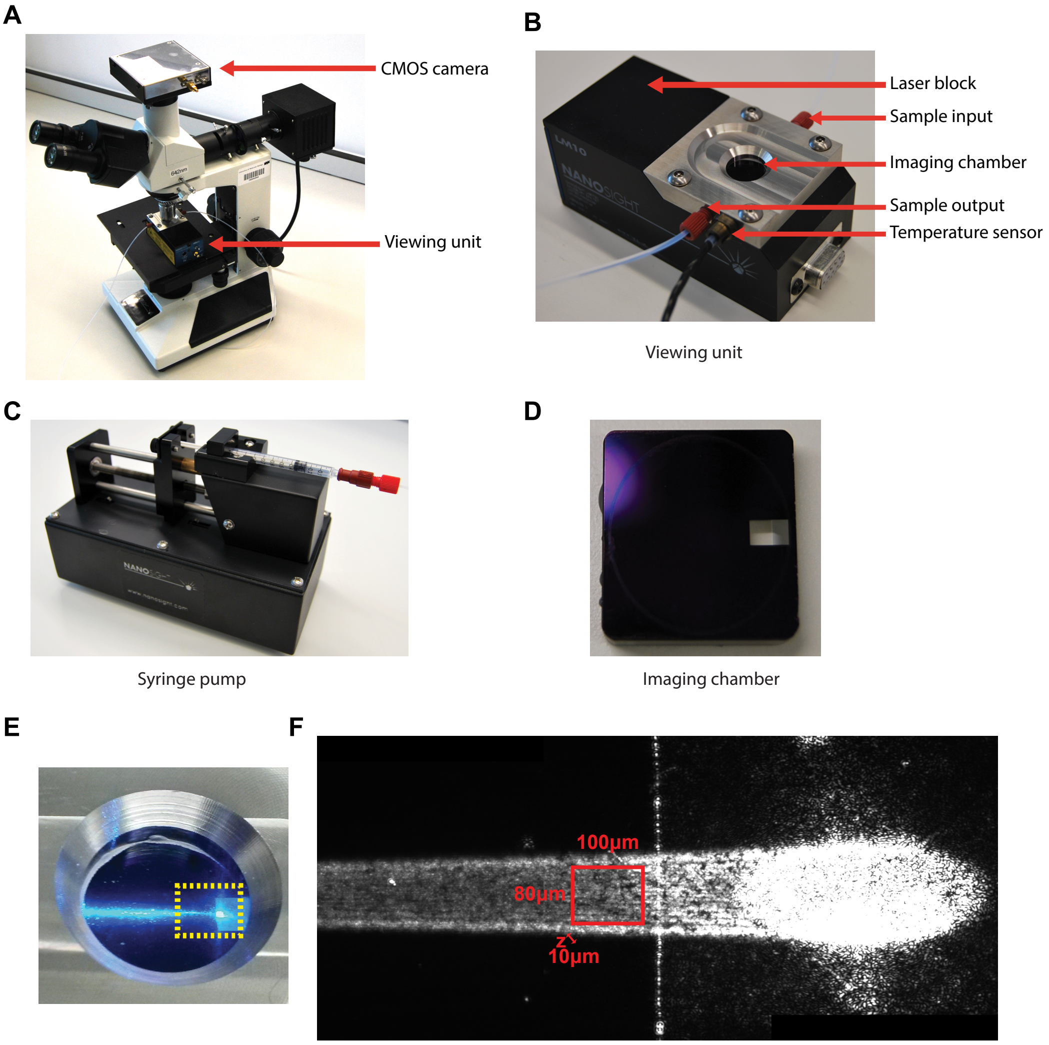

Fig. S1. Overview of the Nanosight instrument.

(A) The NanoSight LM10 (Malvern), used in this study is based on a specially designed viewing unit mounted on a conventional upright microscope with a long working distance 20x objective and equipped with a high sensitivity CMOS camera. **(B)** The viewing unit consists of an imaging chamber (12mm Ø) mounted on a laser block. The unit has ports for sample in/output and for the temperature sensor. **(C)** The sample is injected into the viewing unit using a syringe pump and imaged in a **(D)** specially designed glass chamber where the bottom surface, called the optical flat, is coated with a metallised surface to reduce background. **(E)** Light from the integrated laser passes through the non-coated window in the optical flat and gets refracted upon reaching the liquid sample, forming a beam through the sample. In (**F**), we show a low magnification view of the flow chamber using a 4x objective. The flare on the right represents the point at which the laser exits the optical flat, the vertical line to its left is the boundary between the coated and uncoated glass surface. Measurements are taken in an 80 µm x 100 µm x 10 µm observation volume (indicated by the red box) next to the boundary line on the

Journal of Cell Science • Supplementary information

coated surface, where the laser is brightest. As the laser beam goes through the sample, it quickly loses brightness. Measurements consist of 120 s movies recorded using the high-sensitivity CMOS camera operating at 25 frames per second.

Journal of Cell Science • Supplementary information

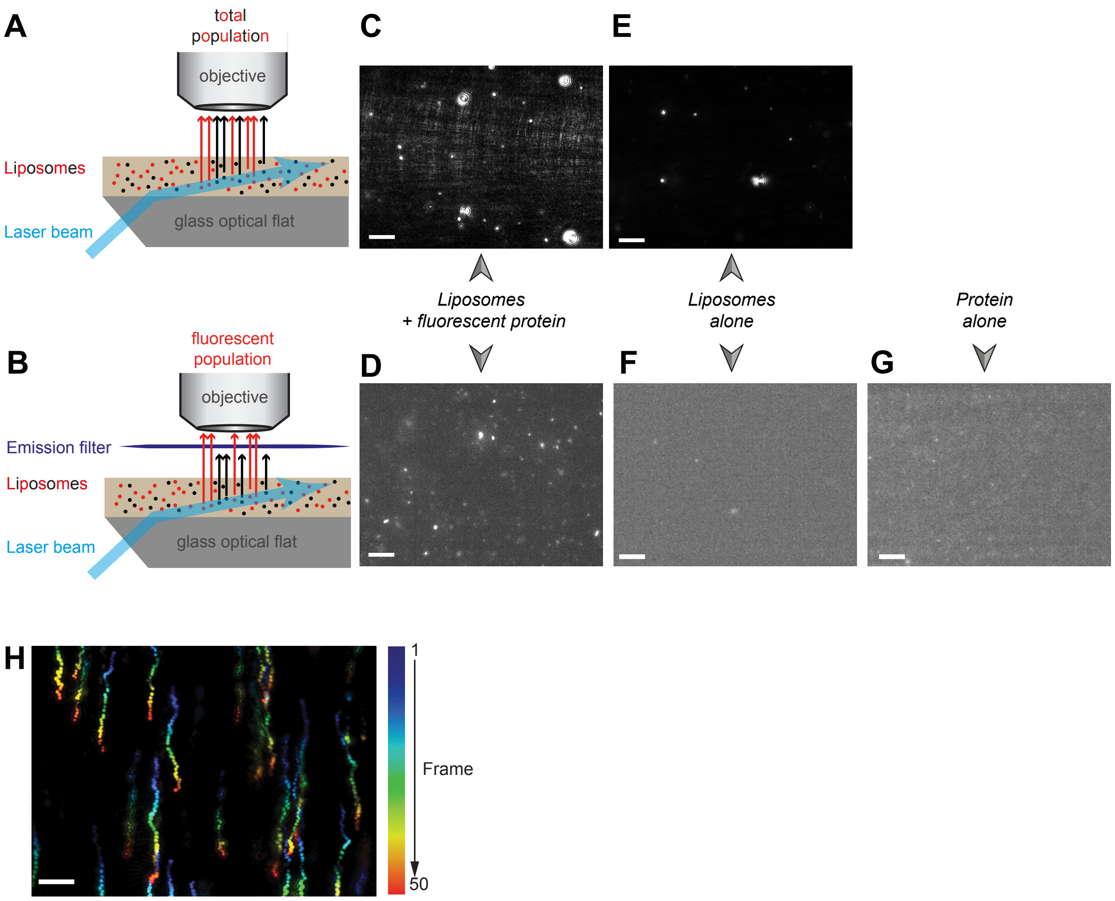

Fig. S2. Measurements with the Nanosight instrument.

Particles are detected using diffraction of the incident laser **(A,C)**, whereas protein-bound liposomes are identified as fluorescent particles using a long pass emission filter **(B,D)**. The lack of background fluorescence derived from liposomes is illustrated by comparing images of a sample of regular liposomes detected using diffraction **(E)** and under fluorescence **(F)**. In addition, signal from protein alone (here FchO2 as an example) is also shown **(G)**. After background subtraction, the centre of mass of each particle is determined. The threshold for detection of particles can be adjusted depending on the intensity of the particles present in the sample. Particles are then automatically tracked and their size calculated based on their Brownian motion. The average displacement of all particles across the field of view is measured and used to subtract the flow generated by the pump in the Brownian motion calculation. The algorithm used for particle tracking is described in ISO 19430:2016. Panel **(H)** shows an example of temporally colour-coded particles moving along the field of view. Although fluorescence can be used to detect particles, quantifying its intensity to calculate the fluorescent-protein coverage on each liposome is limited by intrinsic characteristics of the machine. The most important limiting factor for fluorescence measurement is that the illumination is not even across the Nanosight field of view. As can be seen in Figure S1F,

Journal of Cell Science • Supplementary information

illumination varies both in “x”, along the axis of the laser beam, due to scattering of the light as the laser crosses the sample as well as in “y” due to imperfect glass surface where the laser is diffracted. Moreover, as particles move freely in three dimensions and are imaged in widefield mode, movements in z will also result in varying detected intensities as particles out of focus will still appear on the image and have a clear centre of mass to be able to be tracked. In addition, fluorescent signals decay as the particles flow along the chamber due to bleaching. Scale bar = 10μm

Journal of Cell Science • Supplementary information

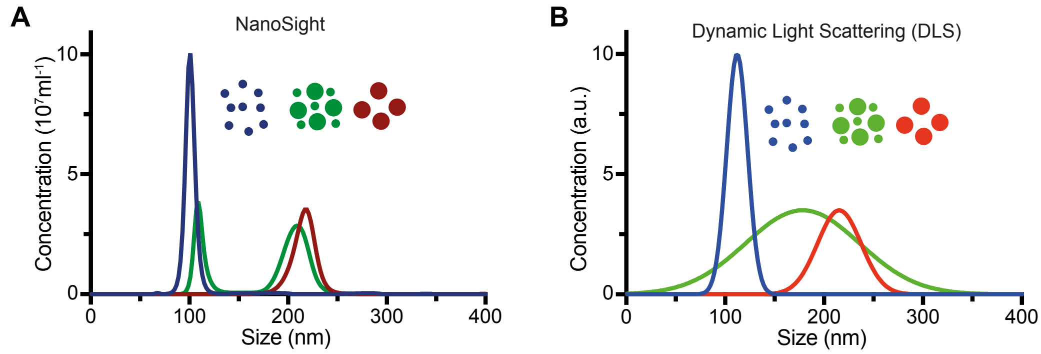

C

beads	NanoSight	DLS
100nm	100±5nm	110±10nm
216nm	217±11nm	214±22nm

beads	NanoSight	DLS
Mix of 100, 216nm	2 peaks 109±5nm 208±13nm	177±58nm

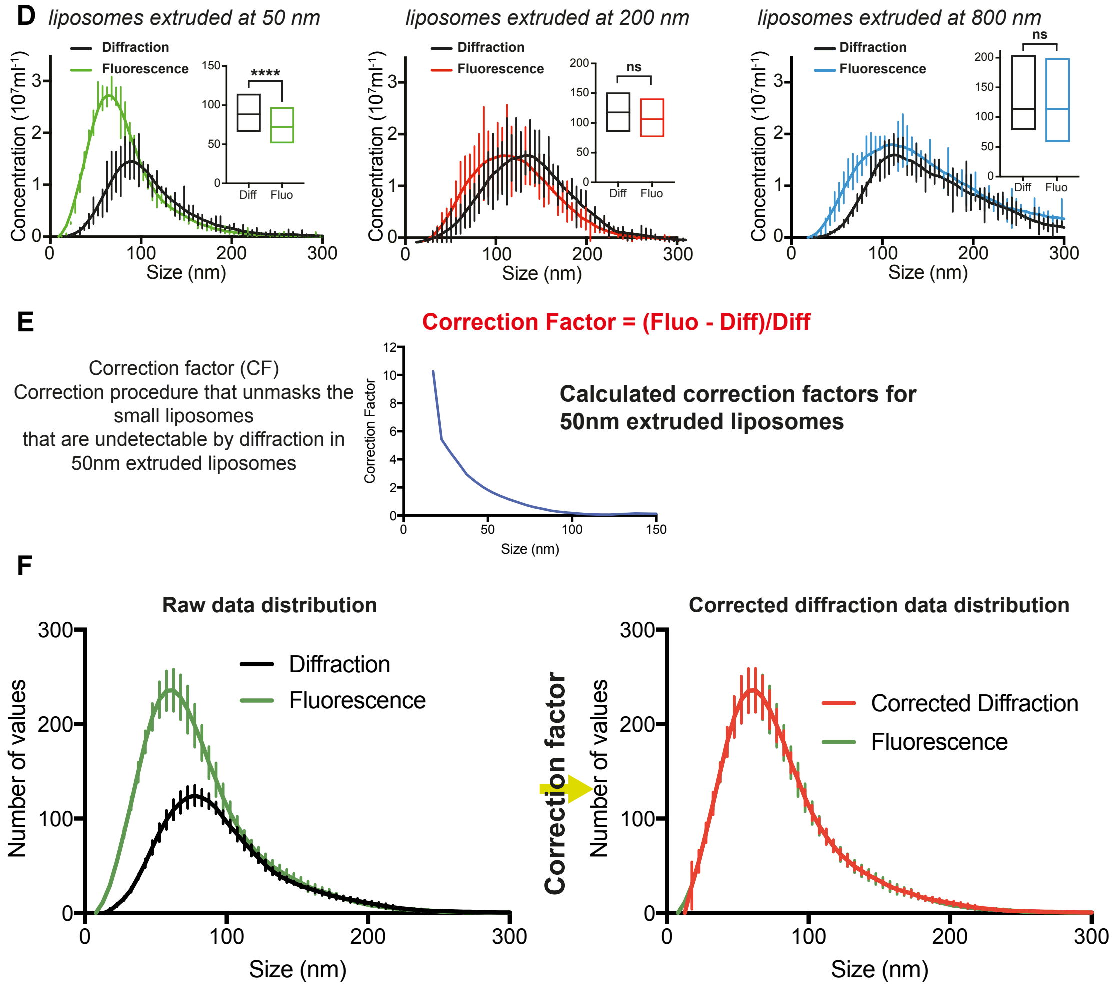

Fig. S3. Data processing, visualisation and correction

A-C. Comparison of sizing of 100 nm (blue), 216 nm (red) beads or a mix (green) by NanoSight (A) or DLS (B) and corresponding bead size measurements (C). Results are indicated as mean ± standard deviation of bead diameters.

D. Comparison of size distributions of fluorescent liposomes using diffraction or fluorescence measurements shows that smaller liposomes are better detected by fluorescence than by diffraction. This is especially obvious with liposomes extruded at 50 nm. Boxplots depict mode values (middle line) -/+ 50% data on each side of the mode (indicated by bottom and top lines). n =3, ns = non-significant, p<0.0001 (****). Two tailed Student's t-test.

E-F. To correct for the non-detectable liposomes from measurements using diffraction, a correction factor is applied. Application of the correction factor on the diffraction data (f, right, black curve) results in a corrected diffraction size distribution (f, left, red), which overlaps well with the size distribution obtained by fluorescence (f left, green).

Journal of Cell Science • Supplementary information

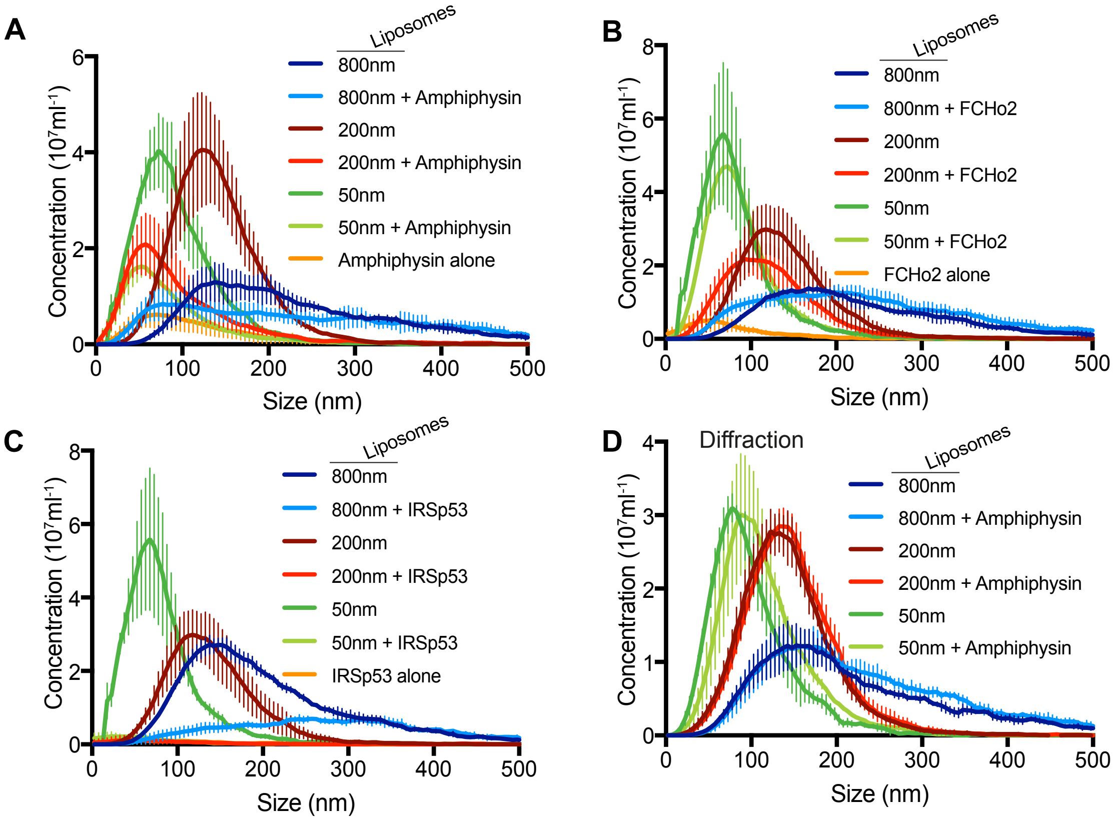

Fig. S4. Raw size distributions for measurements using BAR domains

A-C. Size distributions of liposomes extruded at 800 nm (dark blue), 200 nm (dark red) or 50 nm (dark green) with corresponding size distributions of these liposomes detected by the presence of bound protein (light blue, light red, light green) using Amphiphysin N-BAR (A), FCHo2 F-BAR (B) or IRSp53 I-BAR (C). Signal from protein in the absence of liposomes is shown in orange.

D. Size distributions of the total liposome population (detected by diffraction) in the absence (darker colours) or presence (lighter colours) of Amphiphysin N-BAR shows that no large-scale remodelling of liposomes occurs.

Journal of Cell Science • Supplementary information

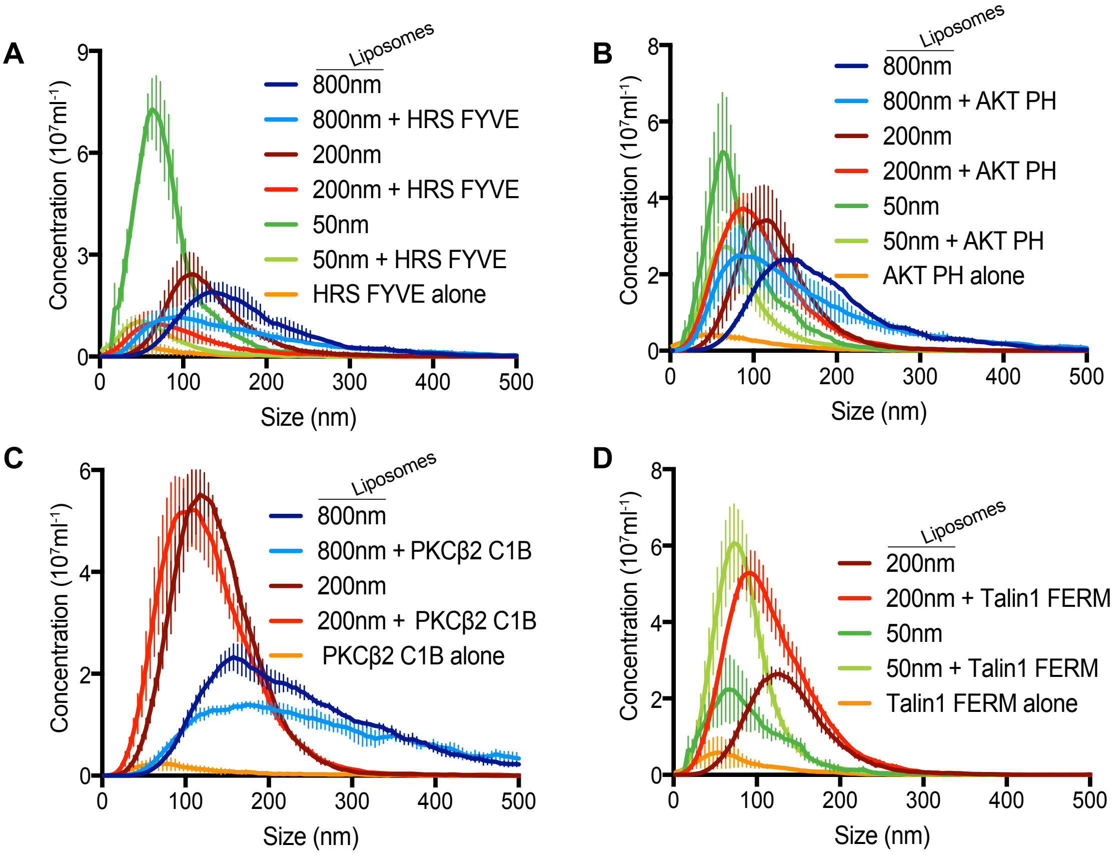

Fig. S5. Raw size distributions measurements of diverse lipid-binding domains

A. Size distributions of liposomes extruded at 800 nm (dark blue), 200 nm (dark red) or 50 nm (dark green) with corresponding size distributions of these liposomes detected by the presence of bound HRS FYVE (light blue, light red, light green). Signal from HRS FYVE in the absence of liposomes is shown in orange.

B. Size distributions of liposomes extruded at 800 nm (dark blue), 200 nm (dark red) or 50 nm (dark green) with corresponding size distributions of these liposomes detected by the presence of bound AKT PH (light blue, light red, light green). Signal from AKT PH in the absence of liposomes is shown in orange.

C. Size distributions of liposomes extruded at 800 nm (dark blue) or 200 nm (dark red) with corresponding size distributions of these liposomes detected by the presence of bound PKCβ2 C1B (light blue, light red). Signal from PKCβ2 C1B in the absence of liposomes is shown in orange.

D. Size distributions of liposomes extruded at 200 nm (dark red) or 50 nm (dark green) with corresponding size distributions of these liposomes detected by the presence of bound Talin1 FERM (light red, light green). Signal from Talin1 FERM in the absence of liposomes is shown in orange.

Journal of Cell Science • Supplementary information

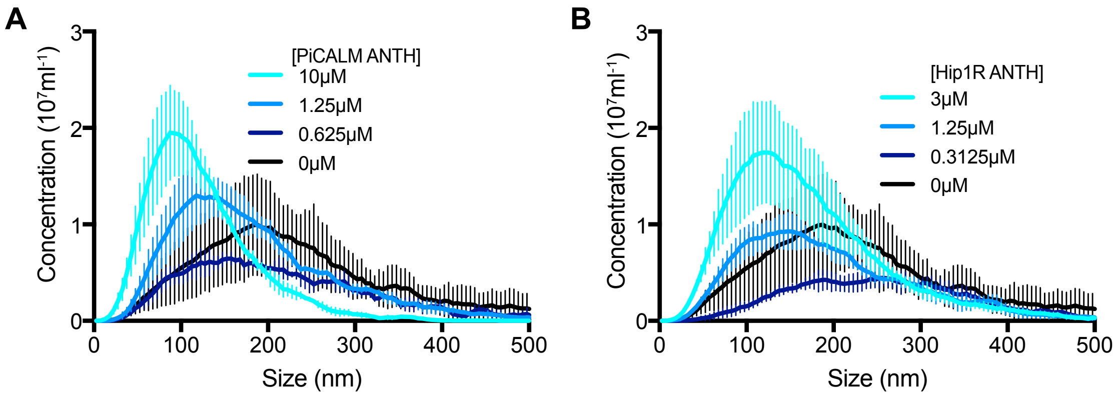

Fig. S6. Detecting membrane vesiculation

A, B. Size distributions of liposomes showing a dose dependent vesiculation by PiCALM ANTH (A) and Hip1R ANTH (B).

Journal of Cell Science • Supplementary information

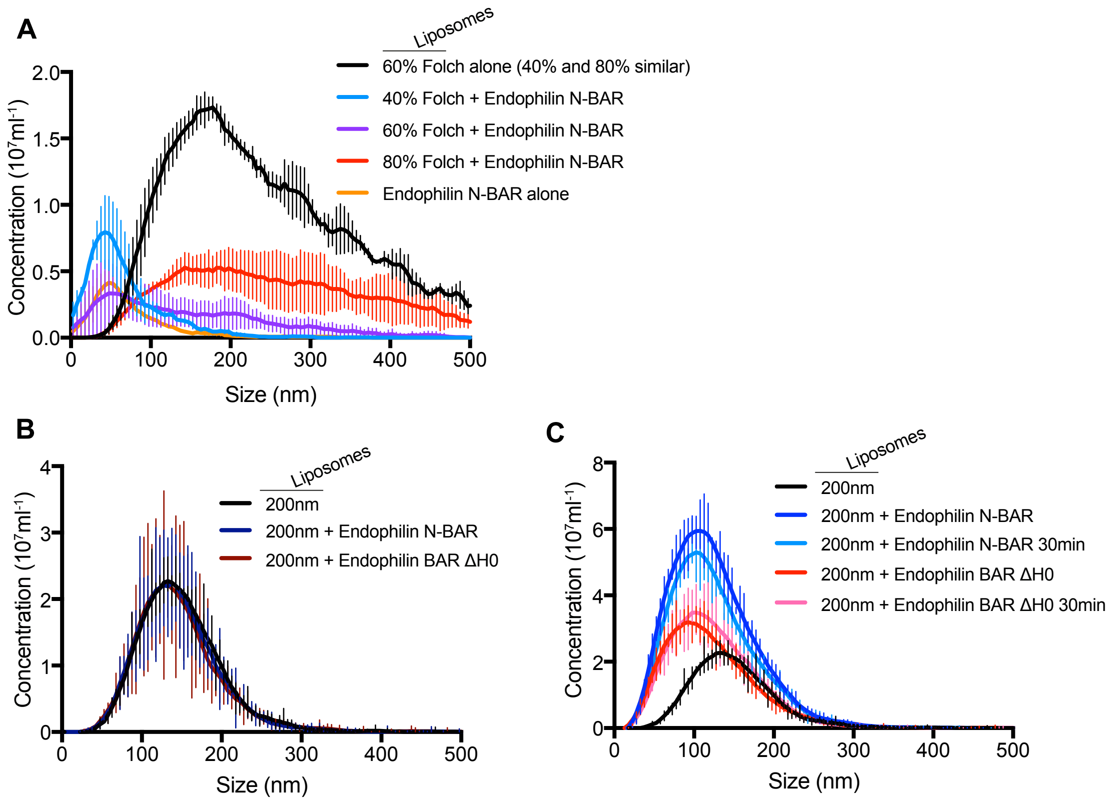

Fig. S7. Mechanism of curvature sensing by Endophilin

A. Raw size distributions for measurements of Endophilin N-BAR-GFP on liposomes with increasing concentrations of Folch lipids.

B. Similar size distributions of liposomes before (black) and after addition of Endophilin N-BAR (blue) or Endophilin BAR ΔH0 (red) shows that no vesiculation is taking place.

C. Longer incubation (30 minutes) with Endophilin N-BAR (light blue) or BAR ΔH0 (pink) does not change the size of the protein-bound liposomes compared to immediately after mixing protein and liposomes (dark blue for Endophilin N-BAR, red for Endophilin BAR ΔH0) indicates that neither of the protein vesiculates liposomes.

Journal of Cell Science • Supplementary information

Table S1. Results from the screening of lipid binding domains

Domain	Gene	Lipid composition	Binding	Curvature sensing
**PH**	AKT1[33]	FolchS + 2% PIP_3	+	High curvatures
**PTB**	DAB2[34,35]	FolchS + 2% $PI(4,5)P_2$	+	Not tested
**GRAM**	OXR1	FolchSA	-	Not applicable
**C1**[36]	PRKCB2	FolchS + 1% PMA	+	Curvature insensitive
**C2**	PLA2G4A[37]	POPC (+ $CaCl_2$)	-	Not applicable
**C2**	SYT1[38,39]	FolchSA + 10% $PI(4,5)P_2$ or PIP_3 (+ $CaCl_2$)	-	Not applicable
**FYVE**[28,40]	HGS	FolchS + 2% PI(3)P	+	High curvatures
**FERM**[41]	TLN1	FolchS + 2% $PI(4,5)P_2$	+	Curvature insensitive

Journal of Cell Science • Supplementary information
